# Interventions to restore appropriate immune function in the elderly

**DOI:** 10.1186/s12979-017-0111-6

**Published:** 2018-01-25

**Authors:** Richard Aspinall, Pierre Olivier Lang

**Affiliations:** 1 Rivock Ltd, Bury St Edmunds, UK; 2 Geriatric and Geriatric Rehabilitation Division, Department of Medicine, University Hospital of Lausanne, Lausanne, Switzerland; 3 Anglia Ruskin University, Cambridge, UK

## Abstract

Advanced age is one indicator of likely immune dysfunction. As worldwide, the global population contains progressively more and more older individuals there is likelihood of an increased prevalence and incidence of infectious diseases due to common and emergent pathogens. The resultant increase in mortality and morbidity would be matched by the risk of functional decline and disability. Maintaining immune function at a plateau throughout life may therefore be associated with considerable cost savings. The aim of improving immune function in older individuals may be achieved through considering a therapeutic approach to rejuvenate, stimulate or support the indigenous immune system to perform in a more optimal manner. In terms of cost effectiveness a therapeutic approach may prove difficult because of issues associated with; identifying those who would benefit the most from this treatment, identifying the type of treatment which would suit them and identifying whether the treatment was successful. The alternative of supporting or providing a stronger stimulus through vaccination, whilst more cost effective, may be a more valuable option in the short term. Both approaches will be addressed in this review.

## Background

The slow and inexorable increase in the number of older individuals worldwide over the next few decades will have a considerable impact on healthcare services and also on the epidemiology of transmissible and non transmissible diseases. The latter are expected to reach unprecedent rates [1, 2]. Those over the age of 65 currently travel more frequently and more widely than either their parents or grandparents [3]. They are also physically more active than their counterparts a few decades ago and these factors will play a role in changing the epidemiology of disease. Another factor contributing to the problem is the way the globe is now so closely networked that any individual or any pathogen may cross the planet within hours, as has been reported recently with H5N1, H1N1, MERS, SRAS, Chikungunya, and other emerging pathogens outbreaks [4, 5]. Also we must include into this algorithm the increased vulnerability in part, due to the decline in immune functioning in this older group. All together, these factors will contribute to shift in the pattern of common and emerging infectious diseases.

Pre-emptive action is required to preserve this growing sector of the general population and to keep them functionally independent in their daily lives. Whilst vaccination is one of the most effective medical interventions ever introduced and prevents millions of cases of infections worldwide every year, vaccines are often thought to be less effective in providing protection in this older section of society. One major reason for this statement is again the decline seen in effective immunity in this population. Studies from several countries have reported that the immune system declines with age indicating the global nature of this problem, but there are fewer studies which have sought the solution to this problem. One approach has been to try to restore immunity within this population to something akin to that seen in younger individuals. Another has been to take a more practical approach believing that a weaker immune system may be provoked into providing a response if the stimulus is considerably strengthened and enhanced. Both approaches will be discussed in this review.

## Recipients

Identification of individuals who would receive specific therapy to restore their immune function is very challenging. The main issue is to how to recognise an individual who is not immune competent enough to deal with new and/or common antigens but who still seems healthy. This has been approached in a very pragmatic manner by some policy makers. To the outsider, it would appear that decisions have been made in the following way

(i) Immune senescence or immune insufficiency is associated with old age.
(ii) Individuals are considered old when they reach a specific age, frequently thought of as around 60-65 in developed and developing countries because after this age people receive benefits and concessions.
(iii) Since individuals who are over 65 are old they must have dysfunctional immune systems.
(iv) So everyone over 65 should be offered vaccines which are supplemented with adjuvants or increased amounts of antigen to compensate for immune decline.

Our issue with such a dogmatic approach is with the idea of using precisely defined criteria, which in this case is age, with immune dysfunction which is imprecisely defined and only weakly linked with the ageing process. What we believe to be required is a means of grading immune functioning quantitatively and qualitatively [6].

## Appropriate immune functioning

One of the problems with the immune system is that its action is invisible and hence not easy to quantify. The immune system provides protection from a series of potential pathogens which we may encounter daily. Their failure to cause disease is neither recognised nor perceived. We have no way of counting the rate of our exposure to pathogenic organisms and we only become aware of the role of the immune system in our survival when it is lacking or through the incidence of some specific diseases. Of course, individuals who are immune deficient show an increased susceptibility to opportunistic pathogens, poorer responses to vaccination, and greater likelihood of morbidity and mortality associated with infections. However, infections are not restricted to those individuals, and healthy people with normal functioning immunity also suffer from viral, bacteria, fungal and parasitic infections.

As a consequence, and in comparison to organs such as the skin where there is greater visibility of age-related changes and methods for measuring them, neither device nor method exists for assessing an individual’s overall immune capacity and its rate of change. People can be identified after vaccination with for example influenza antigen whose responses are less than adequate making them liable to infection with the corresponding virus strains. But this is one of a few cases following vaccination where immunity is associated with an a priori defined amount of specific antibody. Clinicians wishing to measure immune competency may call for full blood counts, T and B cell counts including their subsets, the quantity of immunoglobulin in the serum and the presence of specific antibodies. Whilst this provides a general overview of some of the elements of the immune system it does not provide a functional measure of the capacity of an individual to respond to a specific threat unless values are well below the normal boundaries. An older individual with immune parameters within the normal levels who may have immune dysfunction would thus not be easily identified. So any attempt to restore immunity in older individuals first requires that simple methods of assessment are derived to determine the effectiveness of the process as a whole. These assessment methods must (i) be function related; (ii) must be relatively rapid in providing results; (iii) must be relatively non-invasive; and (iv) require relatively simple forms of equipment.

In view of these guidelines it becomes apparent that such measures as improved responses to vaccination (against for example untreated age matched controls) or reduction in the incidence of infection may be acceptable as measures in the long-term but in the short-term there needs to be some more immediate indicators.

## Contributing factors to immune decline

Change in the fat content of tissues varies between individuals and over the life course. Overall, the body composition changes with age and there is a gradual loss of lean body mass combined with the accumulation of fatty tissue at different locations [7, 8]. Nowhere is this more apparent than in the primary lymphoid organs where the increase in adipocyte numbers leads to macroscopic changes in the colour of these tissues, and the loss of cellular niches and a gradual decline in their overall function as primary generator of lymphocytes. Reduction in the active area of thymus through fat accumulation leads to a loss of an adequate productive environment for thymocyte development and a consequent reduction in the thymic output [9–11]. Similarly the increase in the amount fat in the bone marrow with age as shown by its change in colour from red to yellow alters the ability of the marrow to provide a supportive framework for the production of B cells [12]. In addition to change in the rate of production of new lymphocytes, immune decline may also be linked to the longevity of specific lymphocyte clones. This is a complex process; since lymphocytes have a limited replicative lifespan and the faster more of them reach the limit through responding to antigen the more rapidly will be the onset of functional holes in the repertoire and an inability to respond to a specific challenge.

Adipose tissue expansion with age is also thought of as a major source of inflammation that has a marked influence on systemic metabolism and contributes to the onset of devastating conditions such as insulin resistance, type-2 diabetes, and cardiovascular disorders [13]. However, during the development of diet-induced obesity or age-associated adiposity, immunocompetent cells infiltrate and proliferate in the adipose tissue. Cells of the adaptive immune system, including T and B cells, thus contribute significantly to generate chronic and lowgrade inflammation. Lymphocytes regulate recruitment of innate immune cells into fat tissue, and hence produce a large panel of pro-inflammatory cytokines [14]. This influences the helpful-to-harmful inflammatory balance that, in turn, contributes to the age-associated functional decline in the immune system and the onset of conditions affecting health and well-being [15].

## Approaches to restoration or stimulating immune function

In considering older individuals we may choose to improve their immunity specifically, non-specifically in the periphery, or consider rejuvenating their immune system through the reversal of the decline observed in their primary lymphoid organs. Each of these will be dealt with in turn.

### Restoration through enhanced stimulation or metabolic manipulation

#### Nutrition

One of the issues which is often overlooked is the considerable amount of energy required to undertake an immune response. Naïve T cells which are normally in a close to quiescent state must shift to activation by changing their metabolism after encountering antigen. Successful activation is followed by an increased uptake of nutrients, increased mitochondrial oxidative phosphorylation and the cells metabolism becomes predominantly glycolytic. This shift presages the demand for energy and cell components precursors required for proliferation [16]. For many older individuals the necessary energy to make a successful immune response may be lacking because of either an inadequate diet or the change in the permeability of the gut to specific elements. Insufficient energy to deliver an immune response which is adequate may be due to a number of simple reasons. For example one previous study indicated a significant association between inadequate calorie intake and being underweight in older people with deficient or poorly functioning dentition [17]. Studies in which the diet of older individuals was supplemented, with an energy source and trace elements revealed that treated individuals could produce a greater response to influenza [18–21] and pneumococcal vaccination [22, 23]. Changes in the permeability of the gut alter the uptake of trace elements such as zinc. The critical role of zinc for the immune system comes from studies on zinc deficient individuals who may have thymic atrophy, lymphopenia and recurrent infection [24] however studies on zinc supplementation of the diet of older individuals has failed to provide a conclusive result which would be beneficial to vaccine practitioners [25]. The immunomodulatory role and the potential immune enhancer property of vitamin D have been highlighted recently. Studies which have looked at ability of vitamin D supplements to improve the effectiveness of vaccines provide conflicting information [26].

#### Rapamycin

The fungus *Streptomyces hygroscopicus* found in a soil sample from Easter Island was shown to produces a macrolide compound named rapamycin which was subsequently found to inhibit proliferation of mammalian cells and to possess immunosuppressive properties. These properties led to it being approved by the Food and Drug Administration in the United States for cancer therapy such as renal cell carcinoma. The normal oral dose for cancer patients would be in the region of 10 mg a day. Later experiments in mouse models suggested that at lower doses rapamycin could improve response to influenza antigen possibly by inhibiting the formation of germinal centres thus reducing the production of high affinity antibodies but permitting the production of lower affinity antibody and increasing the presence of influenza-specific IgM antibodies [27]. These results prompted trials in humans where recipients received 0.5 mg daily, or 5 or 20 mg weekly or placebo. After a six-week course and a two-week gap the recipients were vaccinated with the trivalent influenza vaccine. Those in the treatment group showed considerable and significant improvement in their immune response when compared to those in the placebo group [28].

#### Adjuvants

One approach to restoring an effective immune response to a vaccine antigen in older individuals and to ensure adequate levels of protection has been to introduce an adjuvant in the vaccine composition [29]. The classic adjuvant containing aluminium salts has more recently been replaced by oil in water emulsions which have been used with great effect to promote a response to a zoster subunit vaccine for example. However success with adjuvants is a double edged sword. In one study of those who received the adjuvanted herpes zoster subunit vaccine 81.5% showed injection site reactions and 66.1% showed systemic reactions [30]. The mechanisms of action of adjuvants are not entirely clear yet. It is thought that they may in part act as depots of antigen prolonging it presence in the periphery, or interact with pattern recognition elements on accessory cells, or stimulate a local inflammatory response so bringing more cells to the area [29]. Whilst adjuvants have been shown to be effective in improving vaccine related responses in older adults their use may be limited to vaccines which are given infrequently. For example it may not be wise to vaccinate an older individual every year for 30 years (from age 65 to 95) with an influenza vaccine supplemented with adjuvant. Used in such a way this may drive all specific clones of responding cells to reach their replicative limit sooner which may prove counterproductive.

#### Increased amounts of antigen

Increasing the amount of antigen within the dose of vaccine has been also shown to improve the response in older individuals, but not in a directly correlative manner. So, giving for example ten times the antigen dose will not produce a ten-fold increase in the immune response to the corresponding vaccine antigen. Vaccines with increased amounts of antigen include the influenza vaccine with four times the amount of antigen than the standard-dose vaccine (60 μg instead of 15 μg of haemagglutinin antigen for each of the three influenza A/H1N1, A/H3N2 and B strains) and Zostavax a live attenuated varicella zoster virus vaccine administered only to seniors, which contains more than 14 times the number of plaque forming units than the standard varicella vaccine recommended for children. This latter vaccine boosts immunity to the varicella zoster virus in older individuals and significantly reduces the incidence of herpes zoster and post-herpetic neuralgia [31].

### Specific rejuvenation

#### Adoptive transfer of specific clones

One of the issues which is often considered to be a major problem in older people is latent virus infections. Control of these viruses is present throughout life but as we age this control begins to wain as seen with those harbouring the varicella zoster virus (human herpes virus 3 or HHV-3) who become susceptible to increased bouts of shingles the disease associated with herpes zoster and an increase in the prevalence of post herpetic neuralgia with age. Control of cytomegalovirus (CMV or HHV-5) has also been shown to decline with age with the appearance of detectable virus in the urine of older people [32]. Studies using the adoptive transfer of CMV specific CD8 T cells showed that overt disease could be controlled in individuals post-transplant [33, 34]. Whilst this treatment is still at an early phase and unlikely to be offered to older individuals in the near future more recent work suggests that virus specific T cells could be generated from autochthonous stem cells [35].

### Non-specific rejuvenation

#### Blood transfusion

Heterochronic parabiosis; the surgical joining of two animals of different ages such that they have a common blood circulation has been used successfully in the past to identify means of ameliorating age related changes in the cardiac system [36] and in cognitive function [37]. Experiments to modify the immune system of the older partner of the parabiont by an extrinsic factor from the younger parabiont revealed that whilst pre-T cells from either parabiont could seed the opposing thymus and undergo thymopoiesis there was no reversal of thymic atrophy suggesting that the defect was in the thymic environment [38, 39]. Immune rejuvenation by a serum based factor is therefore ruled out as an option but the idea of rejuvenating the peripheral immune system by the adoptive transfer of blood from younger to older individuals has a long history. In the early 1920’s Prof A.A. Bogdanov tested the hypothesis that the transfusion of blood from young into older individuals may rejuvenate the latter. He experimented on himself, accepting transfused blood on up to 11 occasions, before the 12th transfusion killed him in 1928 [40].

Half a litre of blood (500 ml) is the amount normally donated for transfusion and this amount of blood will contain approximately 7.5 × 10^8^ T cells. In individuals aged 20 to mid 30’s approximately 50% or more of these cells will be naïve and the rest memory cells. It would seem feasible to collect this amount of blood and extract the leukocytes on a regular basis and then store them in ampoules in liquid nitrogen until the individual becomes much older. The memory cells will have a repertoire dependent on the pathogens and antigens already encountered and so should be slightly more restricted in terms of its diversity than the naïve T cell population. The latter should contain recent thymic emigrants and so have a diverse receptor repertoire.

In older individuals age related changes in both the peripheral T and B pool of cells include the presence of holes in the antigen specific receptor repertoire which consequently is diminished and the presence of lymphocytes at or close to their limit of replication. Re-transfusion of leukocytes at a later stage of life could provide a means of combatting both of these problems. There are of course issues around how many cells to transfuse back into a recipient, whether this number would integrate easily into the indigenous population and whether the receptors for specific pathogens would be present in the transfused lymphocytes. Preliminary answers to these questions have been provided by animal work [41], but more work is needed on human volunteers to determine these parameters. Despite this, several companies have grown up offering a service for individuals to collect their leukocytes early in their lifespan and store them in lymphocytes in liquid nitrogen for a prolonged period so that they can be reinfused at later date.

#### Interleukin 7

Interleukin 7 (IL-7) is a type 1 short chain cytokine produced by the stromal cells of primary and secondary lymphoid organs which binds to a cell surface receptor composed of an IL-7 specific alpha chain (CD127) and a common gamma chain (CD132). The latter is termed common since it is used as one of the receptor chains for cytokines IL-2, 4, 9, 15 and 21 [42]. Expression of CD127 and CD132 together are found at several stages of the T cell development pathway from early progenitors to memory cells. At the early intrathymic stages of the T cell pathway the IL-7:IL-7R interaction is responsible for ensuring cell survival [43] may be responsible for generating T cell receptor diversity [44] and is associated with clonal expansion of mature thymocytes prior to their export to the naïve T cell pool [45]. In peripheral T cells the IL-7 ligand receptor interaction is mainly associated with clonal maintenance through ensuring cell survival and proliferation in both the naïve and memory T cell pools [46]. The potential for IL-7 to act as an immune rejuvenating agent came from initial studies by Bhatia et al. [47] on young mice treated for prolonged periods with anti-IL-7 antibody. These mice showed severe thymic atrophy and a decline in thymic cellularity similar to that seen in older animals. Treatment of old mice with IL-7 could reverse age-related atrophy of the thymus, leading to a restoration of thymic output and an improvement in peripheral T cell function [43, 48]. One striking feature of using IL-7 to reverse thymic atrophy was the importance of the quantity of IL-7 present in the thymus as shown by experiments in which three lines of transgenic mice were generated in which the IL-7 gene was placed under the control of an lcx promoter. Each line produced different amounts of IL-7 and the line which produced the most (termed TgB) showed lower levels of thymocytes numbers when compared with wild type controls and were found to have a bottleneck in differentiation at the early stage of T cell development [49]. Later work suggest that high levels of IL-7 antagonise Notch signalling and as such impact on the choice of T versus B cell lineage by stem cells in the thymus [50]. Rejuvenation of immunity in old female rhesus macaques treated with recombinant simian IL-7 prior to vaccination with influenza showed an increase in thymic output and higher haemagglutinin titres in treated animals compared to saline treated controls [51]. The first clinical trial using IL-7 examined the therapeutic effects of IL-7 administered to humans with metastatic cancer [52]. A more recent placebo controlled double blind phase IIa trial in patients with lymphopaenic metastatic breast cancer [53], some of whom received recombinant IL-7 as part of their treatment regimen reported encouraging results with IL-7 inducing a significant increase in T cell numbers when delivered before chemotherapy. In another study on HIV infected patients who were also receiving antiviral therapy the data suggest that repeated cycles of recombinant human IL-7 were tolerated quite well by the recipients and sustained T cell restoration was seen in the majority of the participants of the study [53]. To date no trial has reported on the treatment of healthy older individuals who have received IL-7 therapy.

#### Surgical or chemical castration

Early work on rodents showed that surgical castration could lead to reversal of the age associated atrophy seen in the thymus [54, 55] although this has been reported to be transitory [56]. Chemical castration using luteinizing hormone releasing hormone agonists have been shown to be effective in inducing thymic regrowth in old rodents [57] and in old primates [58] through a mechanism which may involve the upregulation of Delta-like 4 a Notch ligand crucial for T cell differentiation [59]. Experiments in humans suggest that this might be an attractive approach to improve immunity in older males [60, 61], however the side effects associated with the treatment may mitigate against this choice of approach.

#### Keratinocyte growth factor

Keratinocyte Growth Factor (KGF) is a member of the fibroblast growth factor family, a family which contains secreted proteins which signal to receptor tyrosine kinases and intracellular non-signalling proteins which act as co-factors for voltage gated calcium channels [62]. KGF is the 7th member of this family and a secreted signalling molecule which interacts with its receptor expressed on thymic epithelial cells and induces the proliferation of thymic epithelial cell progenitors and mature epithelial cells in fetal thymic organ cultures [63]. Early experiments in murine system suggested that the subcutaneous administration of KGF to mice for 3 consecutive days at 5 mg/Kg per day enhanced thymopoeisis for almost 2 months. Moreover the treatment restored thymopoeisis in old mice [64]. These results prompted experiments in primates which were reconstituted with CD34^+^ peripheral blood progenitor cells after irradiation. Animals receiving treatment with KGF showed increased frequencies of naïve T cells in their lymph nodes compared with controls and more cells recognised as recent thymic emigrants. In addition, KGF treated animals preserved their thymic architecture up to at least 12 months after the reconstitution [65].

Palifermin is a recombinant form of KGF of bacterial origin which has been used to treat patients receiving chemotherapy or radiation therapy to stimulate the growth of cells in the mucosal barrier. The positive non-human primate results prompted experiments using Palifermin on patients with HIV-1 infection who were receiving antiretroviral therapy. The hypothesis was that poor CD4 levels in these patients was due to reduced thymic function and treatment with Palifermin should improve the blood profile of CD4 cells in these patients. However a randomised double blind placebo controlled trial in HIV infected patients receiving ART [66] revealed no significant change in either the numbers of recent thymic emigrants, naïve cell numbers or thymic size. The dose range used in these studies was from 20 to 60μg/Kg which is considerable less than the 5 mg/Kg given to the mice in the original model and may be the reason why no change was seen alternatively it is possible that the structure of the thymus was such that it was irreparable within the time frame of the experiment.

## Methods of assessment

To illustrate the problems associated with assessing overall immunity let us consider the example of blood pressure management. Years of gathering and analysing data from blood pressure measurement has enabled physicians to derive a normal range for blood pressure values for specific ages and/or specific health status. Thus different thresholds have been tailored according to the best risk/benefit ratio to initiate lowering blood pressure drugs for robust, frail and terminally ill individuals. Specific target values under treatment have been also personalized [67]. No such scheme currently exists for the immune system. Moreover, the challenge to face with the immune system is it’s degree of complexity and predicting individual immune capacity and responsiveness to specific antigen using a single and robust method able to distinguish between a robust, frail and deficient system would be very challenging [68].

### Using vaccine response as a direct method of assessment

The assessment of an individual immune responsiveness by confronting the immune system with common or new vaccine antigens and hence quantifying the quality of the immune functioning in its whole is a desirable challenge. For example, the response to influenza vaccine is blunted in older people so one method of determining the effectiveness of a rejuvenation/restoration therapy has been to carry out randomised clinical trials comparing a treated aged group with a placebo treated control group and assessing the difference in the titre of the hemagglutination inhibition antibody levels. This approach was used successfully in the rapalogue trials [28], but has the drawback that large numbers of individuals have to be recruited for each arm to ensure statistically significant results are achieved.

Longitudinal studies in which the same individual is followed and tested before and after treatment may appear more preferable but has the problem in that if vaccination is used as an assay system it would have to be given before and the after the treatment. This may prove a problem especially when the repeat vaccination is against previously used strains because high pre-vaccination hemagglutination antibody inhibition titres because the history of previously encountered influenza subtypes impacts the ensuing immune response [69–71].

Equally well, with other vaccines such as pneumococcal polysaccharides vaccines subsequent repetitive vaccination would fail to show benefit because of the hyporesponsiveness [72] seen on revaccination. Another option is to choose to issue the participants with illness diaries which monitor the number of infections during the period of the study and also their duration [73]. Compliance is often a factor with this approach as is veracity. Subjective assessment whilst sometimes necessary may not be optimal which is why many have chosen a more objective approach and taken to using indirect methods of assessment.

### Indirect methods of assessment

Clinicians can have access to a large battery of biological tests. They include complete blood counts, lymphocyte subsets, serum immunoglobulin levels and the presence of specific antibodies, none of which could be used to assess immune rejuvenation satisfactorily. This is principally because these values can be similar in individuals who are immune competent and those who provide a less than adequate response. Approaches to identifying improvements in global immunity have included measuring change in the numbers of recent thymic emigrants [51], the presence of increased numbers of naïve lymphocytes or changes in subsets cells at different stages of differentiation [74], cutaneous skin responses [75] or changes in the parameters [76] of the immune risk phenotype [77].

## Conclusion

The issue at the core of the question about whether we can rejuvenate the immune response is one of measurement. We currently have no method of measuring the overall immune capacity of an individual. Often this measure is through clinical observation that the patient has infections which are unusual, persistent, or alternatively which have become recurrent or have progressed to becoming systemic. These are often subjective assessments and there are no objective laboratory based tests which provide a measure of overall immune capacity. This despite a long history of assays which quantify a response to an antigen through demonstrating the antibody titre or assessing an individual T cell specific response to a viral glycoprotein. We only know the level of response associated with protection from a disease for a small handful of pathogens.

There is no normal range for immune activity and until we can provide a method for measuring this activity we have firstly no means of determining whether an individual is in need of therapy and secondly what the effect of that rejuvenation therapy may be. The complexity of the problem is also added to by the identification that a successful immune response is not only dependant on producing enough specific antibody of effector T cells. Susceptibility to disease is also reliant on a number of innate immune system barriers, such as the integrity of the skin, the flushing action of tears, saliva or urine, the action of ciliated epithelium and mucous as well as the response from neutrophils, macrophages and natural killer cells.
